# Supplementary material for: MiR-155-5p and MiR-203a-3p Are Prognostic Factors in Soft Tissue Sarcoma
Source: Cancers (Basel). 2020 Aug 12;12(8):2254. doi: 10.3390/cancers12082254 (PMC7463991; doi:10.3390/cancers12082254)
Supplement: Supplementary file 1 [file cancers-12-02254-s001.pdf]

cancers

MDPI

Article

MiR-155-5p and MiR-203a-3p are Prognostic Factors in Soft Tissue Sarcoma

Thomas Greither [1,*], Franziska Koser [2], Hans-Jürgen Holzhausen [3], Antje Güttler [4], Peter Würl [5], Matthias Kappler [6], Sven Wach [7,†] and Helge Taubert [7,†]

1 Center for Reproductive Medicine and Andrology, Martin Luther University Halle-Wittenberg, 06120 Halle (Saale), Germany; thomas.greither@medizin.uni-halle.de
2 Institute of Physiology II, University of Muenster, 48149 Münster, Germany; Franziska.koser@ukmuenster.de
3 Institute of Pathology, Martin Luther University Halle-Wittenberg, 06120 Halle (Saale), Germany; Hans-juergen.holzhausen@medizin.uni-halle.de
4 Department of Radiotherapy, Martin Luther University Halle-Wittenberg, 06120 Halle (Saale), Germany;. antje.hahnel@medizin.uni-halle.de
5 Department of General and Visceral Surgery, Hospital Dessau, 06847 Dessau-Roßlau ,Germany;. peter.wuerl@klinikum-dessau.de
6 Department of Oral and Maxillofacial Plastic Surgery, Martin Luther University Halle-Wittenberg, 06120 Halle (Saale), Germany; matthias.kappler@uk-halle.de
7 Clinic of Urology and Pediatric Urology, FA University Hospital Erlangen-Nürnberg, 91054 Erlangen, Germany; helge.taubert@uk-erlangen.de(H.T.); sven.wach@uk-erlangen.de(S.W.)
† both authors contributed equally
* Correspondence: thomas.greither@medizin.uni-halle.de; Tel.: +49 345 557 5264

Supplementary

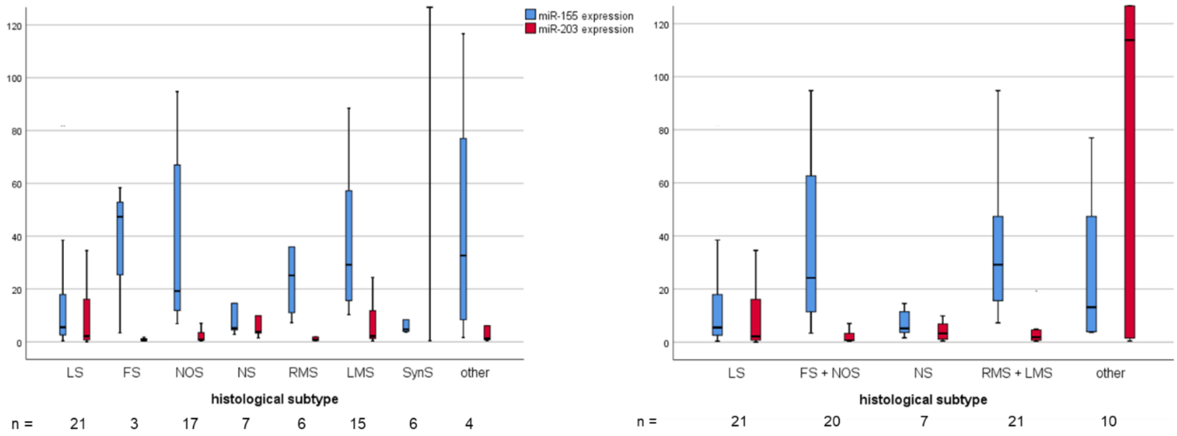

Figure S1. The distribution of miR-155 expression and miR-203 in relation to the histological subtype. (a) Unprocessed and (b) clustered histological subtypes of the analyzed STS patients cohort were analyzed. MiR-155 expression was significantly correlated with the histological subtype (p = 0.01, Kruskal-Wallis test).

cancers

MDPI

Table S1. Univariate and multivariate Cox regression survival analyses of the impact of miR-155 and miR-203 on soft tissue sarcoma patient survival in different histological subtypes. Multivariate Cox regression analyses adjusted for tumor stage, resection type and tumor localization. RR = relative risk with 95% confidence interval in brackets.

	**Univariate Cox regression analysis**			**Multivariate Cox regression analysis**			
**Parameters**	***n***	***p***	**RR**	***n***	***p***	**RR**	
miR-155 low	17		Reference	17		Reference	**liposarcoma (*n* = 21)**
miR-155 high	4	0.449	1.89 [0.36–9.8]	4	0.543	N/A	
miR-203 low	7	0.339	2.1 [0.46–9.61]	7		Reference	
miR-203 high	14		Reference	14	0.311	N/A	
miR-155 low + miR-203 high	10	N/A	N/A	10	N/A	N/A	
miR-155 high + miR-203 low	0		Reference	0		Reference	
miR-155 low	7		Reference	7		Reference	**fibrosarcoma + MFH (*n* = 20)**
miR-155 high	13	0.298	2.04 [0.54–7.46]	13	0.675	1.37 [0.31–6.02]	
miR-203 low	11	0.653	1.33 [0.39–4.54]	11	0.912	1.12 [0.76–8.03]	
miR-203 high	9		Reference	9		Reference	
miR-155 low + miR-203 high	4		Reference	4		Reference	
miR-155 high + miR-203 low	8	0.486	1.53 [0.46–5.03]	8	0.614	1.486 [0.32–6.93]	
miR-155 low	9	0.44	1.52 [0.53–4.36]	9	0.016	13.2 [1.6–106.9]	**rhabdo-/leiomyosarcoma (*n* = 21)**
miR-155 high	12		Reference	12		Reference	
miR-203 low	7	0.1	2.45 [0.84–7.15]	7	0.028	7.16 [1.23–41.6]	
miR-203 high	14		Reference	14		Reference	
miR-155 low + miR-203 high	5		Reference	5		Reference	
miR-155 high + miR-203 low	3	0.54	1.52 [021–4.9]	3	0.43	3.0 [0.19–47.3]	
